# The type 2 acyl-CoA:diacylglycerol acyltransferase family of the oleaginous microalga *Lobosphaera incisa*

**DOI:** 10.1186/s12870-018-1510-3

**Published:** 2018-11-26

**Authors:** Krzysztof Zienkiewicz, Urs Benning, Heike Siegler, Ivo Feussner

**Affiliations:** 1 Department of Plant Biochemistry, Albrecht-von-Haller-Institute for Plant Sciences, University of Goettingen, 37077 Goettingen, Germany; 2 Department of Plant Biochemistry, Goettingen Center for Molecular Biosciences (GZMB), University of Goettingen, 37077 Goettingen, Germany; 3 Department of Plant Biochemistry, International Center for Advanced Studies of Energy Conversion (ICASEC), University of Goettingen, 37077 Goettingen, Germany

**Keywords:** Diacylglycerol acyltransferase, Lipids, Lipid droplet, *Lobosphaera incisa*, Microalgae

## Abstract

**Background:**

Oleaginous microalgae are promising sources of energy-rich triacylglycerols (TAGs) for direct use for food, feed and industrial applications. *Lobosphaera incisa* is a fresh water unicellular alga, which in response to nutrient stress accumulates a high amount of TAGs with a high proportion of arachidonic acid (ARA). The final committed step of de novo TAG biosynthesis is catalyzed by acyl-CoA:diacylglycerol acyltransferases (DGATs), which add a fatty acid (FA) to the final *sn-3* position of diacylglycerol (DAG).

**Results:**

Genome analysis revealed the presence of five putative DGAT isoforms in *L. incisa*, including one DGAT of type 1, three DGATs of type 2 and a single isoform of a type 3 DGAT. For LiDGAT1, LiDGAT2.1, LiDGAT2.2 and LiDGAT2.3 enzyme activity was confirmed by expressing them in the TAG-deficient yeast strain H1246. Feeding experiments of yeast transformants with fatty acids suggest a broad substrate specificity spectrum for LiDGAT1. A significant TAG production in response to exogenous ARA was found for LiDGAT2.2. Cellular localization of the four type 1 and type 2 DGATs expressed in yeast revealed that they all localize to distinct ER domains. A prominent association of LiDGAT1 with ER domains in close proximity to forming lipid droplets (LDs) was also observed.

**Conclusions:**

The data revealed a distinct molecular, functional and cellular nature of type 1 and type 2 DGATs from *L. incisa*, with LiDGAT1 being a major contributor to the TAG pool. LiDGATs of type 2 might be in turn involved in the incorporation of unusual fatty acids into TAG and thus regulate the composition of TAG. This report provides a valuable resource for the further research of microalgae DGATs oriented towards production of fresh-water strains with higher oil content of valuable composition, not only for oil industry but also for human and animal nutrition.

**Electronic supplementary material:**

The online version of this article (10.1186/s12870-018-1510-3) contains supplementary material, which is available to authorized users.

## Background

As photoautotrophic organisms, oleaginous microalgae can synthesize high-energy compounds using CO_2_ as carbon source, water as electron donor and light as primary energy source. Therefore, these organisms gain considerable attention for a renewable production of biofuels, chemical feedstock or bioplastics [1–3]. Unlike land plants, microalgae increase their population rapidly in most cases by a simple vegetative cell division. They are independent from arable land and can be cultivated in fresh, salt, brackish and wastewaters. Thus, unlike oleaginous angiosperms they do not compete with crop plants for cultivable land, which is currently a major dilemma for global oil expansion [4].

Under environmental stress microalgae accumulate substantial amount of lipids, mainly in the form of triacylglyerols (TAGs) [5]. The presence of three fatty acids (FAs) in one TAG molecule results in an extremely high potential energy. However, our knowledge on how lipid metabolism is regulated in most microalgal cells is scarce. Most of the current models rely mostly on the homology between the genetic equipment governing the core pathways of lipid metabolism revealed by recent progress in exploring of a few land plant and microalgal model organisms [6, 7]. However, many aspects of lipid synthesis, accumulation and degradation in microalgal cells seem to be substantially different from the land plants.

As reviewed in [5], in the most general view de novo TAG synthesis in microalgal cells may include the following events: 1) De novo FA biosynthesis in the plastid, 2) Export of either free or bound FAs to the cytosol and their integration into the acyl-CoA pool, 3) Transport of some acyl-CoAs to the endoplasmic reticulum (ER) and their elongation and/or desaturation into long chain (LC) or very-long chain (VLC) polyunsaturated fatty acids (PUFAs), 4) Formation of new lipid molecules within the ER membrane by attaching acyl-CoA molecules in two sequential steps to glycerol-3 phosphate-(G3P) known as Kennedy pathway or by exchanging them with FA from existing lipid molecules, respectively. The Kennedy pathway involves the action of specific ER-membrane bound enzymes. Acyl-CoA:glycerol-3-phosphate acyltransferase (GPAT) catalyzes the formation of lysophosphatidic acid (LPA), which is later acylated at the *sn-2* position by acyl-CoA:lysophosphatidic acid acyltransferase (LPAAT) to form phosphatidic acid (PA). In the next step, PA is dephosphorylated into diacylglycerol (DAG) by phosphatidic acid phosphatase (PAP). This DAG may then either serve as substrate for membrane lipid biosynthesis by the attachment of phospholipid head groups or in case of TAG biosynthesis it is converted by acyl-CoA:diacylglycerol acyltransferase (DGAT), which transfers the third FA to the *sn-3* positon of DAG to form TAG [8, 9]. Newly synthesized TAG molecules accumulate within the leaflet of the ER membrane bilayer, which finally leads to lipid droplet (LD) formation. When budding off from the ER, mature LDs consist of the core composed mainly of TAG and sterol esters surrounded by a single phospholipid monolayer, with a few embedded specific proteins. Recent evidences strongly suggest that LDs are not a simple storage form of cellular lipids but function as dynamic organelles involved in many aspects of cellular metabolism and development [10–13].

DGAT is considered as rate-limiting enzyme of TAG synthesis and accumulation in animals, plants and microbes [14]. Two ER membrane bound DGAT isoforms have been identified in eukaryotes. DGAT of type 1 was initially discovered in mice (MmDGAT1) and comprises of a large number of transmembrane helices [8]. Homologs of DGAT1 genes were discovered in many other eukaryotes including land plants and microalgae [5]. In *A. thaliana* DGAT1 is the main enzyme involved in TAG biosynthesis in seeds [15, 16]. The second type of DGAT (DGAT2) was first identified in the oleaginous fungus *Morteriella ramaniana* [17]. MrDGAT2 contains a MBOAT domain with 1–2 predicted transmembrane helices and was later identified in many other eukaryotes [18]. In land plants, DGATs of type 2 were found to be responsible for the incorporation of unusual FAs into TAG and producing TAG profiles distinct from that of DGAT1, suggesting different substrate specificities between the two DGAT types [8, 19, 20]. Recently, a third type of DGAT was identified in land plants. DGAT3 contains no MBOAT domain and is a soluble protein, making DGAT3 different from DGAT1 and DGAT2 [21]. Though very little is known about DGAT3, it is proposed to be a part of cytosolic TAG synthesis pathway and FA recycling when seed oil breakdown is blocked [22].

Research on oleaginous algae as an alternative source of TAG has increased substantially during the last decade, but knowledge on the core mechanisms of TAG biosynthesis in oleaginous algae is still scarce. This refers especially to nitrogen starvation, which is the most common stress factor for triggering the lipid accumulation in microalgae. Moreover, the unique feature of microalgae is usually the presence of multiple copies of DGAT-encoding enzymes in their genomes, which can vary from 2 up to 13 [5]. On one hand, such a rich set of DGAT copies seems to reflect a high potential of these unicellular organisms for lipid accumulation but on the other hand, it may complicate the role of the analysis of DGAT enzymes even more.

The present study aims at shedding more light on the nature and function of membrane bound DGATs encoded by the genome of *L. incisa*. This fresh water oleaginous microalgae was first isolated from a glacier in Japan and named *Myrmecia incisa* to be renamed later to *Parietochloris incisa* in 2005 [23, 24]. Similar to other microalgae, *L. incisa* accumulates TAG in response to abiotic stress with nitrogen deprivation being the most effective trigger of TAG synthesis. Under such conditions, the TAG content increases from 43% up to 87% of total fatty acids (TFAs) and is accompanied by a concomitant increase in the proportion of ARA in TFAs [25, 26]. Additionally, the analysis of TAG in *L. incisa* revealed high levels of ARA in this lipid class (20:4n-6) [27, 28]. The incorporation of such a PUFA into TAG is rather unique among green microalgae, thus it draws attention to *L. incisa* as the potential source of precursors for high-value lipid products [26]. In view of such a potential, the knowledge on TAG synthesis and especially on DGAT function in *L. incisa* is crucial. However, so far there are only a few reports focusing on DGATs from this microalga. Previous in silico analyses of *L. incisa* transcriptome data revealed 3 isoforms of DGAT based on similarity searches [29, 30]. One DGAT type 1 and two DGAT of type 2 (LiDGAT2.1 (B), 2.2 (A)) were identified and showed to possess DGAT activity. An additional study characterizing the LiDGAT1 enzyme was published recently [31]. In order to understand the function of *L. incisa* DGATs in TAG biosynthesis in more detail, we further analyze the LiDGAT1 and LiDGAT2.1 (LiDGAT2B) and 2.2 (LiDGAT2A), along with a third newly identified putative DGAT2 (LiDGAT2.3). To validate the DGAT activity, the TAG-deficient yeast strain H1246 was complemented with LiDGAT1, LiDGAT2.1, LiDGAT2.2 and LiDGAT2.3 as well as with their double constructs followed by analysis of synthesized TAGs. Additionally, feeding experiments of yeast transformants with FAs were employed in order to get first hints on a potential substrate specificity for all analyzed DGATs. Cellular localization of type 1 and type 2 DGATs from *L. incisa* expressed in yeast was also analyzed to address their cellular behavior.

## Results

### Nitrogen deprivation triggers lipid accumulation in *L. incisa*

**Fig. 1:**
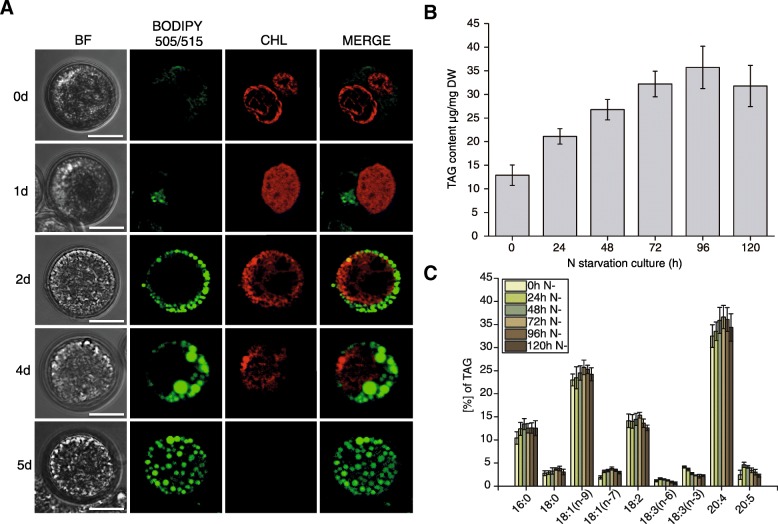
TAG synthesis in *L. incisa* under 5 day-long nitrogen deprivation. **a** Imaging of LDs (green) and chloroplast (red) at subsequent days (d) of culture. Bar = 5 μm. **b** and **c** changes of TAG content (**b**) and FA composition (**c**). BF, bright field; CHL, chlorophyll autofluorescence. Raw data for TAG content and FA composition are given in Additional file 10

Lipid accumulation in *L. incisa* in response to nitrogen deprivation was investigated during a 5 d period of growth in nitrogen free media (Fig. 1). Confocal laser microscopy (CLSM) was used to address the cellular changes occurring in nitrogen deprived *L. incisa* cells (Fig. 1a). During the onset of nitrogen starvation (0 d) no or very few LDs were found, and most of the algal cells contain a single chloroplast as the most prominent organelle. After 24 h of nitrogen stress (1 d), numerous small LDs were detected in most cells. Their number and size increased during further steps of growth under nitrogen-deprived conditions (2d-4d). In parallel, chloroplast autofluorescence gradually diminished throughout the subsequent time-points of growth in absence of nitrogen. At the final analyzed time-point (5 d) a great majority of *L. incisa* cells was entirely filled up with numerous LDs and no chloroplast autofluorescence was observed anymore. These cellular changes were also reflected in the TAG content of *L. incisa* cells growing in absence of nitrogen (Fig. 1b). The content of FAs in TAG increased gradually along with nitrogen starvation in the culture, reaching a plateau at the 4th day followed by a slight decrease at the 5th day. Nitrogen deprivation affected also the profile of FAs in the TAG fraction (Fig. 1c). The most characteristic changes were observed for PUFAs, where the relative amount of 20:4(n-6) gradually increased during first 3 days of culture whereas18:3(n-3) and 20:5(n-3) showed opposite trends, reaching the lowest levels at the 5th day of growth in the nitrogen free medium. The content of monounsaturated 18:1(n-9) and 18:1(n-7) was similar like 20:4(n-6). The relative amount of 16:0 and 18:0 increased during the first two days of nitrogen starvation and reached a plateau during the further analyzed time points (Fig. 1c).

### Five DGATs encoded by the *L. incisa* genome cluster differently within the eukaryotic DGAT family

**Fig. 2:**
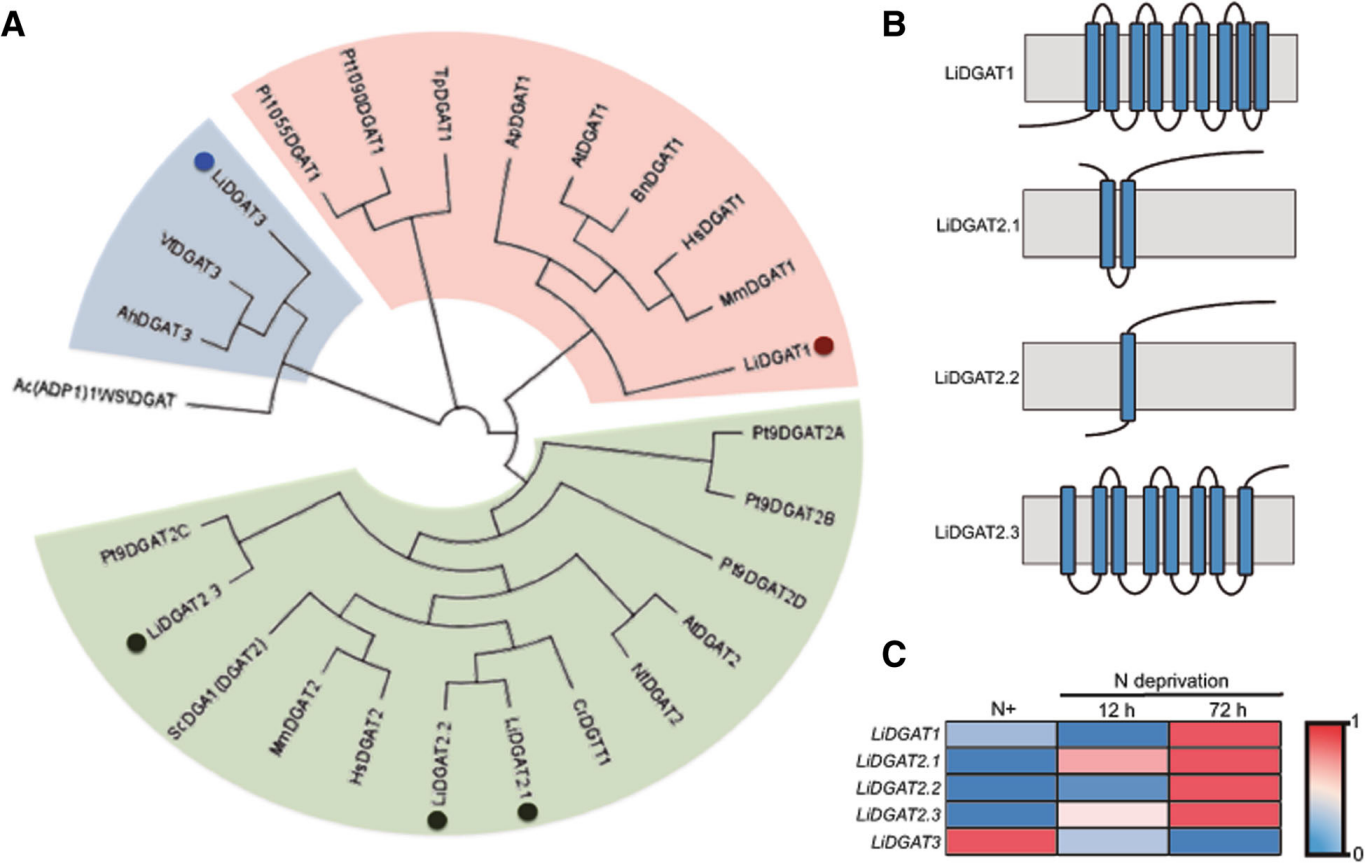
Phylogenetic relationships, topology and expression of LiDGATs. **a** Phylogenetic tree indicating the relationship of LiDGATs (dots) with their animal, plant and algal counterparts (**b**) Topology of transmembrane domains predicted for membrane-bound DGAT proteins of *L. incisa*. **c** Heatmap of normalized expression data for all identified LiDGATs of type 1 and 2 under control and nitrogen starvation conditions. Table listing DGAT-encoding genes used for phylogenetic analyses are given in Addtional file 7. Gene numbers and expression values can be found in Additional file 8

By exploring the *L. incisa* genome [28], we identified five DGAT isoforms. Beside the previously described single copy of type 1 DGAT (LiDGAT1) [31] and two putative DGATs of type 2 (LiDGAT2.1 and LiDGAT2.2) [30], we identified a novel type 2 DGAT, termed as LiDGAT2.3, as well as one putative DGAT3 (LiDGAT3) (Fig. 2a). All the sequences encoding type 2 DGATs of *L. incisa* have been deposited in GeneBank with the following accession numbers: MH290880 (LiDGAT2.1), MH290881 (LiDGAT2.2) and MH290882 (LiDGAT2.3).

Phylogenetic analysis showed that LiDGAT1 clusters with the DGAT1 family, with a closer homology to plant and vertebrate type 1 DGATs than those of diatoms. Nevertheless, LiDGAT1 clusters rather separately from other eukaryotic DGATs of type 1. Among the three isoforms of type 2 DGATs, LiDGAT2.1, and LiDGAT2.2 cluster closely with each other, whereas the novel LiDGAT2.3 clusters away and seems to be more related to one of the type 2 DGATs encoded by the *P. tricornutum* genome. The putative type 3 DGAT from *L. incisa* clusters with plant DGATs of type 3.

To gain more information on the molecular nature of type 2 DGATs from *L. incisa*, we compared their amino acid sequences with DGATs of plants and animals (Additional files 1 and 2). We were able to identify 8 major conserved regions (SPH block, GL block, KSR block, PTR block, QP block, HKW block, FQL block and NGQP block) in the C-terminal part of LiDGAT1. These regions show higher homology to plant rather than animal type 1 DGATs. Interestingly, LiDGAT1 showed also the presence of a very large extra region at the N terminus with no homology to any of the analyzed type 1 DGATs (Additional file 1, N terminal black box). A comparison of the three putative LiDGAT2 isoforms showed their high homology to previously identified [32] C-terminal conserved regions of vertebrate type 2 DGATs, with LiDGAT2.1 and LiDGAT2.2 having higher similarity than LiDGAT2.3 (Additional file 2). In the latter one PH block and YFP block are not fully conserved and the HPHG motive contained instead of the His (H) a Gln (Q).

Further analysis of the structural organization of LiDGAT proteins revealed variations in the number and position of their putative transmembrane domains (Fig. 2b). Nine transmembrane domains were predicted for LiDGAT1, where many of them overlap with conserved regions of other type 1 DGAT. LiDGAT2.1 and LiDGAT2.2 may contain two and one predicted transmembrane domain, respectively. Interestingly, LiDGAT2.3 showed the presence of 8 putative transmembrane domains, but unlike in LiDGAT2.1 and LiDAGT2.2 they not always overlap completely with the conserved regions of members of the DGAT2 family.

The analysis of transcriptomic data shows that all DGATs encoded by the *L. incisa* genome are up-regulated in response to nitrogen starvation, with the exception of LiDGAT3 (Fig. 2c). Their highest expression levels were observed at 72 h of growth in nitrogen free medium [28].

### LiDGATs restore the phenotype of a TAG-deficient yeast mutant to different degrees

**Fig. 3:**
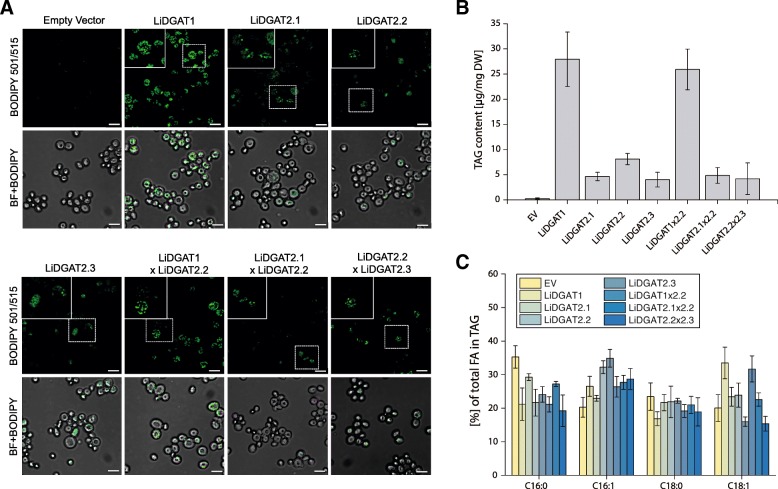
Restoring of TAG synthesis in H1246 yeast mutant by *L. incisa* DGATs heterologous expression. **a** LDs formation analyzed by Bodipy staining (green) in H1246 cells transformed with empty vector and H1246 cells expressing single (LiDGAT1, LiDGAT2.1, LiDGAT2.2 and LiDGAT2.3) and double (LiDGAT1xLiDGAT2.2, LiDGAT2.1xLiDGAT2.2 and LiDGAT2.2xLiDGAT2.3) LiDGAT-encoding constructs. Bars 5 μm. **b** Quantification of TAG levels extracted from transformed yeast showed on (**a**). **c** Changes in the TAG fatty acid profile in response to expression of empy vector (EV) and constructs from (**a**). For experiments showed in (**b**) and (**c**) values represent mean ± SD (*n* = 3). Raw data for TAG content and FA composition are given in Additional file 11

All four putative membrane-bound DGATs encoded by the *L. incisa* genome were transformed into the TAG deficient yeast strain H1246 to test their ability to synthesize TAG. First, the transformed cultures were induced and tested for the expression of the transgene (Additional file 3). The positive lines were then selected for phenotypic analysis (Fig. 3). In addition to the four single constructs (LiDGAT1, LiDGAT2.1, LiDGAT2.2 and LiDGAT2.3), three double constructs encoding LiDGAT1xLiDGAT2.2, LiDGAT2.1xLiDGAT2.2 and LiDGAT2.2xLiDGAT2.3 were also prepared and expressed in the yeast strain H1246 in order to test their potential for restoring the TAG deficient phenotype. Their expression was confirmed in the same way as for the single constructs (Additional file 3).

Screening of yeast transformants on the cellular level showed that the most LDs were formed in the H1246 expressing LiDGAT1 as well as with the tandem construct LiDGAT1 and LiDGAT2.2 (Fig. 3a). The expression of single and double constructs encoding only LiDGATs of type 2 was accompanied by significantly lower labelling by Bodipy 493/505. No labelling was observed in the lines expressing empty vector. Analysis of the TAG content in H1246 expressing LiDGATs correlated well with the microscopic observations (Fig. 3b). Yeast cells expressing the single construct encoding LiDGAT1 or the tandem construct encoding LiDGAT1and LiDGAT2.2 showed the highest TAG content reaching more than 30 μg of TAG per mg of DW. The expression of the single and double constructs encoding only type 2 LiDGATs resulted in TAG levels around 5 μg per mg of DW, with the exception of LiDGAT2.2 having TAG levels of about 10 μg per mg of DW. The analysis of the FA composition of TAG extracted from the transformed yeast revealed differences in the FA profiles between the analyzed lines (Fig. 3c). When compared to the empty vector, LiDGAT1 and LiDGAT1xLiDGAT2.2 produced more18:1 on the expense of 16:0 in the TAG fraction. In turn, the expression of LiDGAT2.2 and LiDGAT2.3 resulted in higher amounts of 16:1 and lower levels of 16:0. Such tendency, however at different levels, was observed for all transformed lines of H1246, when compared to empty vector control.

### Fatty acid feeding reveals diverse preferences of *L. incisa* DGATs

**Fig. 4:**
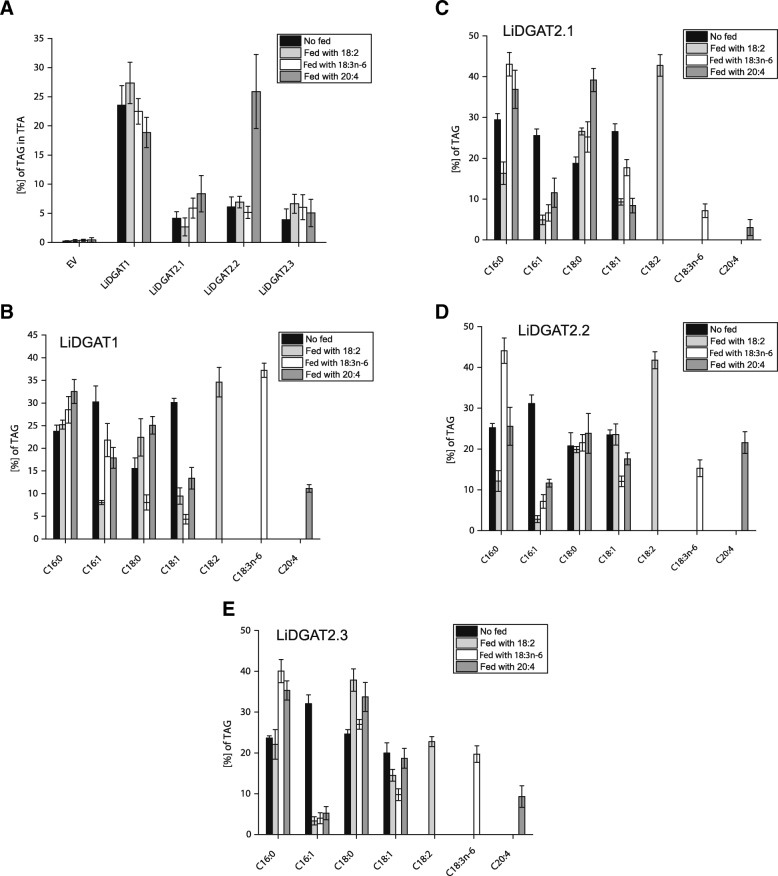
TAG content and composition in yeast cultures in response to expression of LiDGATs and feeding with exogenous FAs. **a** Levels of TAG extracted from yeast expressing empty vector (EV), LiDGAT1, LiDGAT2.1, LiDGAT2.2 and LiDGAT2.3 shown as fatty acids esterified to TAG over total fatty acids (TFA). The cultures were grown in absence (black box) or presence of exogenous 18:2 (light grey bars), 18:3n-6 (white bars) or 20:4 (dark grey bars). **b**-**e** FA profiles of TAG extracted from H1246 transformants showed on (**a**). For all experiments values represent mean ± SD (*n* = 3). Raw data for TAG content and FA composition are given in Additional file 12

To gain more information on the potential substrate preferences of the different LiDGATs, we compared the TAG content and composition of H1246 cells expressing the four single constructs with cultures fed with exogenous FAs (Fig. 4a, Additional file 4). Overall, yeast cells expressing LiDGAT1 showed much higher TAG levels, when compared with type 2 LiDGATs. The only exception was observed for yeast cells expressing LiDGAT2.2 that were fed with 20:4. Here, the TAG content was comparable to that of LiDGAT1 reaching 25.8% of the total FA content.

The analysis of the FA profile of the TAG fraction produced by LiDGAT1-expressing yeast cells showed no obvious preference for any endogenous FA (Fig 4b). When the cells were fed with 18:2 however this FA was incorporated into TAG on the expense of 16:1 and 18:1, while feeding with 18:3n-6 resulted in the reduction of 18:0 and 18:1. When the cells were fed with 20:4, 16:1 and 18:1 were again reduced in the TAG pool. The FA profile of TAG synthesized by LiDGAT2.1 in the absence of exogenous FAs revealed a similar profile to that observed with LiDGAT1 (Fig. 4c). When fed with 18:2, again this FA was incorporated into TAG on the expense of 16:1 and 18:1. Interestingly, incorporation of 18:3n-6 was much lower than that of 18:2 upon feeding and led only to a reduction of 16:1. Feeding with 20:4 resulted in a low incorporation as well as that was accompanied by the reduction of 16:1 and 18:1 in the TAG fraction. FA profiles of the TAG fraction from yeast cells expressing LiDGAT2.2 showed no obvious changes in the FA profile (Fig. 4d). Feeding with 18:2 revealed similar to LiDGAT2.1 the best incorporation for this FA into TAG accompanied by a reduction of 16:1. Feeding with 18:3n-6 led to a moderate incorporation into TAG again on the expense of 16:1 and 18:1. Application of exogenous 20:4 resulted in a similar proportion of all the FA in the TAG fraction. Overall, incorporation of 20:4 was the highest among all analyzed DGATs of *L. incisa*. Analysis of TAG produced by LiDGAT2.3 expressing yeast cultures showed lowest incorporation rates with no preference for one of the three applied FAs (Fig. 4e). Again feeding with exogenous PUFAs resulted mainly in a reduction of 16:1 and to lesser degree of 18:1 in case of feeding with 18:2 and 18:3n-6.

### *L. incisa* type 1 and 2 DGATs show a different cellular localization

**Fig. 5:**
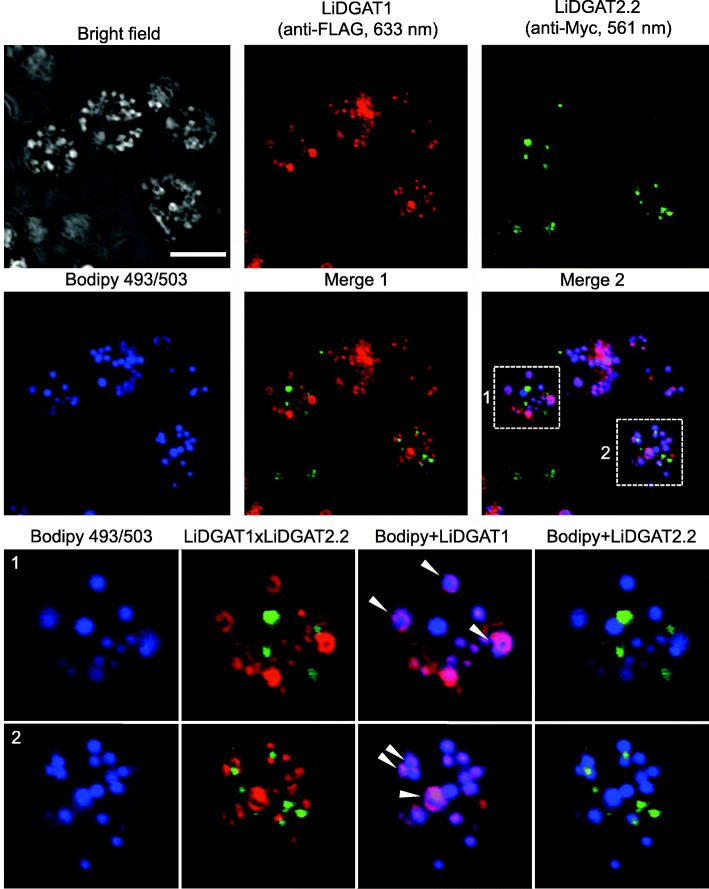
Detection of LiDGAT1 (red), LiDGAT2.2 (green) and LDs (blue) in yeast mutant complemented with the double construct encoding LiDGAT1-Myc and LiDGAT2.2-FLAG fusion proteins by using CLSM. Dashed boxes indicate area magnified in the bottom panel. Arrowheads indicate co-localization of LiDGAT1 with LDs. Bar = 5 μm

Immunodetection of all *L. incisa* DGATs and whole-mount LD staining were carried out to address the cellular localization of these proteins (Additional files 5 and 6, Fig. 5). In yeast cells expressing a LiDGAT1-Myc construct, the majority of the fluorescence signal corresponding to LiDGAT1 was observed in close proximity to LDs (Additional file 5). Such co-localization was not observed for any of the type 2 LiDGATs as the punctate immunofluorescence was present in areas of the cell that were not occupied by LDs. To test if there is any spatial relationship between LiDGAT1 and LiDGAT2.2, a double localization of both proteins was performed. The signals corresponding to LiDGAT1 and LiDGAT2.2 never co-localized. Moreover, the LiDGAT1-derived signal was more prominent than that of LiDGAT2.2 and mostly present in the areas of the cell occupied by LDs (Fig. 5, arrowheads). No co-localization of LiDGAT2.2 in close proximity to LD was found. A control reaction with omission of the primary antibodies did not show any significant fluorescence (Additional file 6).

## Discussion

Our study focuses on type 2 DGAT enzymes of the green microalga *L. incisa*. We identified and characterized here for the first time the third member of this family - LiDGAT2.3 and provide novel data on the molecular, functional and cellular nature of membrane-bound DGATs encoded by the *L. incisa* genome. To achieve that LiDGATs were transformed into the TAG-deficient yeast mutant H1246 to determine their activity and potential synergistic interactions between them.

Nitrogen starvation has been shown to affect algal metabolism by inducing quiescence, re-organization of the photosynthetic apparatus and triggering lipid accumulation [5, 6, 33, 34]. As expected, in the unicellular green microalga *L. incisa*, nitrogen starvation for 7 d (Fig. 1) resulted in a complete degradation of the chloroplast and a massive accumulation of TAG. Similar observations were reported previously for this strain [25, 27, 35]. Beside the ability to accumulate large amount of lipids, *L. incisa* is particularly interesting also in terms of its FA profile, as it shows the highest ARA (20:4) content among plants and algae [27]. Thus, studying its lipid metabolism may add new aspects to our understanding of TAG biosynthesis and even for the incorporation of nutritionally valuable FAs into this fraction.

Five putative DGAT isoforms can be identified in the *L. incisa* genome, of which one was a DGAT1 isoform, three belong to the type 2 DGAT family and one is homologous to plant DGAT3 (Fig. 2). We show here that the protein sequences of LiDGATs are spread over the entire DGAT phylogenetic tree and do not form a distinct group and cluster with sequences from animals, plants or diatoms. As it was shown previously for other microalgal DGATs [36], most of the LiDGATs are more closely related to orthologues in other species than to their paralogues. However, this does not apply to LiDGAT2.1 and LiDGAT2.2, which are closely related to each other. This may suggest that these two genes are the result of an evolutionary recent gene duplication. Overall such a phylogenetic distribution of LiDGATs fits well with the model of a divergent evolution of algal DGATs and suggests that *L. incisa* DGATs might originate from diverse ancestors [5].

The identification of one DGAT1 and two type 2 DGATs from *L. incisa* was previously reported and provided first functional data on these enzymes [30]. Recently a more detailed functional analysis of LiDGAT1 was published [31]. Our transcriptomic data for LiDGAT1 and LiDGAT2.2 fit well with the previously described expression profiles under nitrogen starvation, which reached a maximum at 72 h of growth in nitrogen free medium (Fig. 2c). The newly identified LiDGAT2.3 showed a similar expression pattern, suggesting that all membrane-bound DGATs from *L. incisa* may be involved in TAG accumulation under nitrogen deprivation. However, the expression profile do not match completely with the previously reported ones [30], because in this study only DGAT2A, named LiDGAT2.2 in this study, was responsive to nitrogen starvation and no significant changes in the expression were found for DGAT1 and DGAT2B, named LiDGAT2.1 in this study. For LiDGAT1 it was recently described that upon nitrogen starvation, high light further increases its gene expression [31]. Thus, the differences in expression levels of DGAT genes reported in this study and in [30] could be the result of higher (190 μmol photons m^− 2^ s^− 1^, this study) and lower (115 μmol photons m^− 2^ s^− 1^, [30]) light intensities used for performing the nitrogen starvation experiments. In addition, in the former study q-RT-PCR was used for transcriptional profiling, while our data originate from transcriptome sequencing [28]. Moreover, despite of the evident up-regulation in response to nitrogen starvation the different LiDGAT genes showed different transcript counts (Additional file 2). Therefore, one has to take the light intensity applied during the nitrogen stress experiment into account when comparing DGAT expression profiles. As multiple copies of DGAT-encoding genes are usually present in microalgal genomes [5], differences in their expression in response to nitrogen starvation not only between type 1 and type 2 DGATs but also between specific type 2 DGATs may be a common mechanism to adjust TAG amount and composition [37–40].

The homology analysis of DGAT1 proteins presented here show that LiDGAT1 has a higher similarity to type 1 DGATs from flowering plants than to those of diatoms. Similar conclusions were presented recently [31]. Furthermore, LiDGAT1 was able to successfully complement a TAG-deficient yeast mutant as confirmed by the presence of numerous LDs along with a comparably high TAG content (Fig. 3) and cultures expressing this DGAT incorporated preferentially 18:2 and 18:3n-6 over 20:4 into TAG (Fig. 4). As suggested recently LiDGAT1 can therefore incorporate all major PUFA found in *L. incisa* into TAG [31] and may thus potentially direct the major FA flux into the TAG fraction.

All three type 2 DGATs (LiDGAT2.1, LiDGAT2.2 and LiDGAT2.3) were found to harbor a YFP and a HPHG domain (Fig. 2, Additional file 2, Box I and III). Both motives have been studied in the yeast DGAT ScDGA1 and found to be crucial for DGAT function as mutating them resulted in a loss of enzymatic activity (Liu et al. 2011). Interestingly, out of all the three type 2 DGATs from *L. incisa* only LiDGAT2.2 harbors a fully conserved YFP motive. In turn, the animal-like HPHG motive was only fully conserved in LiDGAT2.1 and LiDGAT2.2, but not in LiDGAT2.3. Similar variations were previously reported for type 2 DGATs of *Chlamydomonas* [41] and *Nannochloropsis* [40] and were proposed to be related to specific catalytic activities and substrate preferences, as it was reported for ScDGA1 and mouse DGAT2 [32, 42].

All analyzed type 2 LiDGATs synthesize TAG and induce LD formation in yeast at similar, though much lower levels when compared to LiDGAT1 (Figs. 3 and 4) and among the type 2 DGATs LiDGAT2.2 was slightly more efficient in TAG formation. The highest incorporation of exogenous PUFAs into TAG was observed for LiDGAT2.2 in case of 20:4, which may suggest that LiDGAT2.2 plays a role in the enrichment of TAG in 20:4 upon nitrogen deprivation. This substrate specificity of within the group of type 2 DGATs in *L. incisa* confirms differences in substrate specificities within algal DGAT2 families previously shown for type 2 DGATs from other green microalgae [40, 41, 43] and diatoms [39].

Interestingly, only LiDGAT2.2 showed a higher specificity for ARA (Figure 4), while the other DGAT2s did not. Moreover, the selectivity for another PUFA, 18:3n-6, was lower for all DGAT2s, compared to DGAT1. This is different from what was reported earlier for Arabidopsis DGAT2 showing a higher selectivity for 18:3n-3 [44]. This may be an evidence for a divergent direction of the evolution of DGAT-encoding genes in land plants and microalgae. In Arabidopsis, there are two membrane-bound DGAT isozymes, one belonging to the class DGAT1 and one to the class DGAT2 genes [15, 44]. Microalgae usually contain multiple copies of DGAT2-encoding genes and their number as well as their phylogenetic relationships are remarkably diverse among algal genera [5]. This diversity is also observed at protein level and results in distinct substrate preferences between DGAT1s and DGAT2s observed not only between higher plants and algae but also between different microalgal strains. Such diversity was observed for instance for DGATs encoded by the genomes of *Chlamydomonas reinhardtii* [41] or *Nannochloropsis oceanica* [40, 45].

To determine whether the LiDGAT isoforms work together, we co-expressed them as tandems in the yeast TAG-deficient mutant (Fig. 3). All tested co-expressions restored the lipid phenotype of the H1246 mutant, however with different efficiency. As expected, co-expression of LiDGAT1 and LiDGAT2.2 resulted in the highest TAG production and number of LDs. However, no additive effects with the analyzed DGATs when expressed in yeast were observed.

Localization of LiDGATs in yeast cells revealed that LiDGATs of type 1 and 2 do not co-localize with each other at least in yeast (Fig. 5), suggesting that they reside in different ER domains. A distinct subcellular localization was previously reported for DGAT1 and DGAT2 from plants [19] and animals [46] and seems to confirm that DGAT1 and DGAT2 may have separate functions [47]. Interestingly, we observed a close spatial relationship between LDs and LiDGAT1,but this may be restricted to yeast cells, since we detected no DGAT in the LD proteome of *L. incisa* in a previous study [28]. Thus, this enzyme seems rather to localize in an ER domain adjacent to LD than being an integral LD protein. Nevertheless, localization of DGATs at the LD membrane has been reported for several animal cells [46, 48]. Moreover, accumulation of type 2 DGAT in ER domains in close spatial proximity to forming LDs was also recently reported for the green microalgae *N. oceanica* [40]. Most likely, such localization of LiDGAT1 reflects its prominent role in TAG accumulation and LD formation. Indeed, recent models from studies in animal cells confirm this important role of type 1 DGATs in LD formation [49]. Together it might suggest such a role for LiDGAT1 in *L. incisa* as well.

## Conclusions

Together with the identification of a novel member of the DGAT2 family in *L. incisa* our data revealed also significant differences between the type 1 and type 2 DGATs from this microalga. Beside their distinct phylogenesis, LiDGATs seem to show distinct preferences towards unsaturated fatty acids, suggesting their diverse role in lipid homeostasis under nitrogen stress. We propose for *L. incisa* that DGAT1 isoforms may contribute mainly to net TAG synthesis and DGAT2 isoforms may play a role in regulating the TAG composition. Namely, LiDGAT2.2 was found to incorporate 20:4 at high rates into TAG and could therefore be the primary target for further studies oriented towards increasing FAs of nutritional value in the TAG fraction. This model is further supported by the localization of LiDGAT1 to LD at least in yeast cells suggesting for a direct involvement of this DGAT in LD formation in *L. incisa*. Overall, our report provides a valuable resource for further studies on microalgal DGATs oriented towards production of fresh-water strains with higher oil content of valuable composition, not only for oil industry but also for human and animal nutrition.

## Material and methods

### Materials

#### Microalgal cultures

*L. incisa* strain SAG 2468 was kindly provided by Dr. Inna Khozin-Goldberg, Ben-Gurion University of the Negev (Israel). It was grown in BG11 medium [50] in 400 mL glass columns with an inner diameter of 3 cm (Ochs GmbH, Bovenden, Germany). Cultures of 300 ml grew under constant light with 190 μmol photons m^− 2^ s^− 1^ at 20 °C and air supplemented with 1% CO_2_. For nitrogen starvation cells were washed and resuspended in modified BG11 media, where NaNO_3_ was omitted and ammonium ferric citrate was replaced with ferric citrate [25]. Nitrogen deprivation was conducted for 5 days and samples were collected every 24 h. For lipid analyses, samples were freeze-dried, subsequently ground in liquid nitrogen and stored − 80 °C. For microscopic studies collected cells were fixed (see below) and stored in 4 °C.

### Sequence and bioinformatic analyses

*L. incisa* genome sequencing was performed as described previously by [28]. For the analysis of the DGAT sequences on the amino acid level, the Geneious 8 software (https://www.geneious.com/) and neighbor joining were used. GeneBank accession numbers of *L. incisa* DGATs and of DGATs used for phylogenetic analysis (Fig. 2) are listed in Additional file 7. Prediction of *LiDGAT* transmembrane domains was performed using TMHMM server 2.0 (http://www.cbs.dtu.dk/services/TMHMM/) using protein sequences given in Additional files 1 and 2.

Transcriptomic data were obtained through GIAVAP transcriptome database (https://giavap-genomes.ibpc.fr/, *P.incisa*_v3.1), normalized and presented as heatmap using Origin Pro 8.5 (https://www.originlab.com/). Raw data for LiDGAT transcript levels are enclosed in Additional file 8.

### Expression of LiDGATs in yeast strain H1246

RNA was isolated from 72 h-long nitrogen deprived culture of *L. incisa* and cDNA was synthesized as described previously by [28]. Coding sequences of interest were amplified and restriction sites were added using the primers listed in Additional file 10A. Amplification was carried out using Phusion High-Fidelity DNA polymerase (Thermo Fisher Scientific) according to the protocol provided by the manufacturer. Amplicons were then ligated into a subcloning vector using the CloneJET PCR cloning kit (Thermo Fisher Scientific) by using the T4 DNA ligase (Thermo Fisher Scientific), following the user manual. After digestion with the appropriate restriction enzymes (Additional file 9A) the coding sequences without a stop codon were then inserted into the respective destination vectors given in Additional file 9B, for expression in H1246 TAG-deficient yeast mutant [51]. Competent cells of H1246 were obtained and transformed according to [52]. Transgene expression was induced by transferring the yeast pre-culture from SD-Ura medium with 2% (*w*/*v*) raffinose after 24 h at 30 °C to SD-Ura medium with 2% (w/v) galactose at OD600 nm 0.2. After 48 h at 30 °C of the cultures were pelleted by centrifugation for 5 min at 3000 g and collected for microscopic studies or stored in − 80 °C for lipid analysis.

#### Feeding experiments

For the feeding experiments, yeast cultures were induced as described above but in presence of 1% (w/v) Tergitol NP-40 (Sigma Aldrich, St. Louis, MO, USA) in the medium. At the beginning of induction, the appropriate FAs were added to the culture to a final concentration of 50 μm. Samples at OD600 of 2.5 were harvested for lipid extraction, separation by TLC and analysis by GC (see below).

### Lipid profiling

Lipids from *L. incisa* cultures were extracted and analyzed as described previously by [28, 53], respectively.

Extraction of lipids from yeast cultures was initiated by adding 1 mL of methanol and 0.5 mm glass beads to the yeast frozen pellet followed by 1 h long-shaking at 4 °C. Next, 2 mL of hexane were added and samples were vortexed for 1 h at 4 °C. After centrifugation at 1000 g the upper phase was collected, evaporated under gas N_2_ and dissolved in 50 μl of acetonitrile (Sigma Aldrich). Lipids were then separated by thin layer chromatography (TLC) with diluted olive oil (1:100 (by vol.) in acetonitrile) as a standard. 50 μL of each sample and 25 μL of standard were spotted onto a TLC silica gel (Millipore Corporation, Billerica, MA, USA) and lipid separation was performed in a solvent mixture composed of n-hexane:ethyl ether:acetic acid (80:20:1, by vol.) for 30 min. Bands containing TAG were scrapped out and transferred to a glass tubes. Preparation, extraction of fatty acid methyl esters (FAMEs) and their analysis by GC with flame ionization detection (FID) was done as described previously by [53].

### Microscopic analyses

#### LD staining

For LD staining in *L. incisa* cells 10 ml of samples were collected at selected time points of nitrogen deprivation, pelleted by centrifugation at 1000 g for 5 min and fixed with 4% (*w*/*v*) paraformaldehyde in 0.1 M PBS, pH 7.4, overnight at 4 °C. Fixed samples were washed three times with the same buffer and stained with 10 μg/ml Bodipy 493/504 (Thermo Fisher Scientific) diluted in 0.1 M PBS buffer, pH 7.4, for 1 h at RT with gentle agitation. Then samples were washed 3 times with the PBS buffer only and re-suspended in Prolong gold anti-fade reagent (Thermo Fisher Scientific). LD staining in yeast cells was performed as described in [40].

#### Immunolocalization of LiDGATs in yeast cells

Immunofluorescence detection of LiDGATs expressed in yeast cells with myc-tag and/or FLAG-tag was carried out according to the protocol of [54]. Mouse monoclonal anti-myc antibody (Sigma Aldrich) and rabbit monoclonal anti-FLAG antibody (Sigma Aldrich) were used as primary antibodies at dilution 1:50 in 0.1 M PBS pH 7.4 with 1% (w/v) bovine serum albumin (BSA). The incubation with one (single localization experiments) or mixture of two primary antibodies (double localization experiments) was carried out overnight at 4 °C. Next day the samples were washed three times with 0.1 M PBS buffer, pH 7.4, and incubated with secondary antibodies, respectively, goat anti-mouse Alexa 633 and/or goat anti-rabbit Alexa 546 (Thermo Fisher Scientific) diluted 1:100 in the same buffer with 1% (w/v) BSA. Incubation with secondary antibodies was carried out for 2 h at RT in the dark with gentle agitation. After washing three times with the PBS buffer samples were suspended in Prolong gold anti-fade reagent (Thermo Fisher Scientific) and immediately analysed with confocal microscope.

#### Confocal microscopy

Images were taken with a LSM 510 Meta Confocal Laser Scanning Microscope (Carl Zeiss Mikroskopie; Jena, Germany) using a 63x/1.40 Plan-Apochromat 1.4 Na oil immersion lens. Bodipy 493/504 and chlorophyll excitation in *L. incisa* cells was achieved using an argon laser and two different channels collected the emission spectra with a wavelength of 500–515 nm and 630–670 nm, respectively. Immunoflurescence analysis in yeast cells was carried out with the use of an argon laser for excitation of Bodipy 493/504 and two diode lasers exciting Alexa 546 (556 nm) and Alexa 633 (663 nm). The emission spectra were collected at 500–515 nm, 560–590 nm and 630–660 nm, respectively.

Analysis and processing of the images was done using the LSM 5 image browser (Carl Zeiss Mikroskopie).

## Additional files

Additional file 1: Alignment of *L. incisa* LiDGAT1protein sequence with its animal and plant counterparts and identification of conserved regions. (PDF 4293 kb)

Additional file 2: Alignment of amino acid sequences of *L. incisa* LiDGATs of type 2 with selected animal and plant DGAT2s and identification of conserved regions. (PDF 3602 kb)

Additional file 3: Expression analysis of DGAT-encoding cDNAs in H1246 yeast mutant. (PDF 410 kb)

Additional file 4: Representative TLC plates showing activity of LiDGATs expressed in yeast mutants without and with feeding with exogenous FAs. TAG bands were used for lipid analysis showed in Fig. 4. (PDF 6144 kb)

Additional file 5: Immunodetection of LiDGAT1 (red), three type 2 LiDGATs (green) and LDs (blue) in H1246 cells complemented with the single constructs. Anti-myc antibody and anti-FLAG antibody were used for detection of LiDGAT1 and each of LiDGAT2, respectively. Bar = 5 μm. (PDF 12506 kb)

Additional file 6: Control reaction of immunodetection of LiDGATs performed with omission of the primary antibodies. No fluorescence corresponding to LiDGAT1 at 633 nm or LiDGAT2.2 at 561 nm can be observed. Labelled LDs are shown in blue. (PDF 4265 kb)

Additional file 7: Table listing DGAT-encoding genes used for phylogenetic and sequence analyses. (PDF 151 kb)

Additional file 8: Raw transcriptomic data on LiDGATs under diverse nitrogen conditions. (XLSX 9 kb)

Additional file 9: List of the vectors used in this work for yeast transformation. (PDF 95 kb)

Additional file 10: Raw data of content and composition of TAG under nitrogen starvation of *L. incisa*. (XLSX 15 kb)

Additional file 11: Raw data from lipid analysis in H1246 expressing single and double constructs encoding LiDGATs. (XLSX 12 kb)

Additional file 12: Raw data from lipid analysis in H1246 expressing single LiDGATs and fed with exogenous FAs. (XLSX 21 kb)

## Abbreviations

ARA: Arachidonic acid
CLSM: Confocal scanning laser microscopy
DAG: Diacylglycerol
DGAT: acyl-CoA:diacylglycerol acyltransferase of type 1
ER: Endoplasmic reticulum
FA: Fatty acid
FAME: fatty acid methyl ester
GC-FID: Gas chromatography-flame ionization detection
LD: Lipid droplet
PUFA: Polyunsaturated fatty acid
ScDGA1: *S. cerevisiae* diacylglycerol acyltransferase 1
TAG: Triacylglycerols
TLC: Thin-layer chromatography

## References

[1] Hempel F, Bozarth AS, Lindenkamp N, Klingl A, Zauner S, Linne U, Steinbüchel A, Maier UG (2011). Microalgae as bioreactors for bioplastic production. Microb Cell Factories.

[2] Stemmler K, Massimi R, Kirkwood AE (2016). Growth and fatty acid characterization of microalgae isolated from municipal waste-treatment systems and the potential role of algal-associated bacteria in feedstock production. PeerJ.

[3] Zeller MA, Hunt R, Jones A, Sharma S (2013). Bioplastics and their thermoplastic blends from *Spirulina* and *Chlorella* microalgae. J Appl Polym Sci.

[4] Carlsson AS, Yilmaz JL, Green AG, Stymne S, Hofvander P (2011). Replacing fossil oil with fresh oil – with what and for what?. Eur J Lipid Sci and Technol.

[5] Zienkiewicz K, Du Z-Y, Ma W, Vollheyde K, Benning C (2016). Stress-induced neutral lipid biosynthesis in microalgae — molecular, cellular and physiological insights. Biochim Biophys Acta.

[6] Zulu NN, Zienkiewicz K, Vollheyde K, Feussner I (2018). Current trends to comprehend lipid metabolism in diatoms. Prog Lipid Res.

[7] Li-Beisson Y, Beisson F, Riekhof W (2015). Metabolism of acyl-lipids in Chlamydomonas reinhardtii. Plant J.

[8] Cagliari A, Margis R, dos Santos Maraschin F, Turchetto-Zolet AC, Loss G, Margis-Pinheiro M (2011). Biosynthesis of triacylglycerols (TAGs) in plants and algae. Internatl J Plant Biol.

[9] Dahlqvist A, Stahl U, Lenman M, Banas A, Lee M, Sandager L, Ronne H, Stymne S (2000). Phospholipid:diacylglycerol acyltransferase: an enzyme that catalyzes the acyl-CoA-independent formation of triacylglycerol in yeast and plants. Proc Natl Acad Sci U S A.

[10] Farese RV, Walther TC (2009). Lipid droplets finally get a little R-E-S-P-E-C-T. Cell.

[11] Jacquier N, Choudhary V, Mari M, Toulmay A, Reggiori F, Schneiter R (2011). Lipid droplets are functionally connected to the endoplasmic reticulum in Saccharomyces cerevisiae. J Cell Sci.

[12] Chapman KD, Dyer JM, Mullen RT (2012). Biogenesis and functions of lipid droplets in plants. J Lipid Res.

[13] Chapman KD, Ohlrogge JB (2012). Compartmentation of triacylglycerol accumulation in plants. J Biol Chem.

[14] Liu Q, Siloto RMP, Lehner R, Stone SJ, Weselake RJ (2012). Acyl-CoA:diacylglycerol acyltransferase: molecular biology, biochemistry and biotechnology. Prog Lipid Res.

[15] Hobbs DH, Lu C, Hills MJ (1999). Cloning of a cDNA encoding diacylglycerol acyltransefrase from *Arabidopsis thaliana* and its functional expression. FEBS Lett.

[16] Zou J, Wei Y, Jako C, Kumar A, Selvaraj G, Taylor DC (1999). The *Arabidopsis thaliana TAG1* mutant has a mutation in a diacylglycerol acyltransferase gene. Plant J.

[17] Lardizabal KD, Mai JT, Wagner NW, Wyrick A, Voelker T, Hawkins DJ (2001). Dgat2 is a new diacylglycerol acyltransferase gene family. purification, cloning, and expression in insect cells of two polypeptides from *Mortierella ramanniana* with diacylglycerol acyltransferase activity. J Biol Chem.

[18] Turchetto-Zolet A, Maraschin F, de Morais G, Cagliari A, Andrade C, Margis-Pinheiro M, Margis R (2011). Evolutionary view of acyl-CoA diacylglycerol acyltransferase (DGAT), a key enzyme in neutral lipid biosynthesis. BMC Evol Biol.

[19] Shockey JM, Gidda SK, Chapital DC, Kuan J-C, Dhanoa PK, Bland JM, Rothstein SJ, Mullen RT, Dyer JM (2006). Tung tree DGAT1 and DGAT2 have nonredundant functions in triacylglycerol biosynthesis and are localized to different subdomains of the endoplasmic reticulum. Plant Cell.

[20] Ayme L, Baud S, Dubreucq B, Joffre F, Chardot T (2014). Function and localization of the *Arabidopsis thaliana* diacylglycerol acyltransferase DGAT2 expressed in yeast. PLoS One.

[21] Saha S, Enugutti B, Rajakumari S, Rajasekharan R (2006). Cytosolic triacylglycerol biosynthetic pathway in oilseeds: molecular cloning and expression of peanut cytosolic diacylglycerol acyltransferase. Plant Physiol.

[22] Hernandez ML, Whitehead L, He Z, Gazda V, Gilday A, Kozhevnikova E, Vaistij FE, Larson TR, Graham IA (2012). A cytosolic acyltransferase contributes to triacylglycerol synthesis in sucrose-rescued Arabidopsis seed oil catabolism mutants. Plant Physiol.

[23] Karsten U, Friedl T, Schumann R, Hoyer K, Lembcke S (2005). Mycosporine-like amino acids and phylogenies in green algae: *Prasiola* and its relatives from the Trebouxiophyceae (Chlorophyta). J Phycol.

[24] Watanabe S, Hirabayashi S, Boussiba S, Cohen Z, Vonshak A, Richmond A (1996). *Parietochloris incisa* comb. nov. (Trebouxiophyceae, Chlorophyta). Phycol Res.

[25] Khozin-Goldberg I, Bigogno C, Shrestha P, Cohen Z (2002). Nitrogen starvation induces the accumulation of arachidonic acid in the freshwater green alga *Parietochloris incisa* (*Trebuxiophyceae*). J Phycol.

[26] Hu Q, Sommerfeld M, Jarvis E, Ghirardi M, Posewitz M, Seibert M, Darzins A (2008). Microalgal triacylglycerols as feedstocks for biofuel production: perspectives and advances. Plant J.

[27] Bigogno C, Khozin-Goldberg I, Adlerstein D, Cohen Z (2002). Biosynthesis of arachidonic acid in the oleaginous microalga *Parietochloris incisa* (*Chlorophyceae*): radiolabeling studies. Lipids.

[28] Siegler H, Valerius O, Ischebeck T, Popko J, Tourasse NJ, Vallon O, Khozin-Goldberg I, Braus GH, Feussner I (2017). Analysis of the lipid body proteome of the oleaginous alga *Lobosphaera incisa*. BMC Plant Biol.

[29] Ouyang L-L, Chen S-H, Li Y, Zhou Z-G (2013). Transcriptome analysis reveals unique C4-like photosynthesis and oil body formation in an arachidonic acid-rich microalga Myrmecia incisa Reisigl H4301. BMC Genomics.

[30] Chen C-X, Sun Z, Cao H-S, Fang F-L, Ouyang L-L, Zhou Z-G (2015). Identification and characterization of three genes encoding acyl-CoA: diacylglycerol acyltransferase (DGAT) from the microalga *Myrmecia incisa* Reisigl. Algal Res.

[31] Sitnik S, Shtaida N, Guihéneuf F, Leu S, Popko J, Feussner I, Boussiba S, Khozin-Goldberg I. DGAT1 from the arachidonic-acid-producing microalga *Lobosphaera incisa* shows late gene expression under nitrogen starvation and substrate promiscuity in a heterologous system. J Appl Phycol. 2018. 10.1007/s10811-10017-11364-10813.

[32] Liu Q, Siloto RMP, Snyder CL, Weselake RJ (2011). Functional and topological analysis of yeast acyl-CoA:diacylglycerol acyltransferase 2, an endoplasmic reticulum enzyme essential for triacylglycerol biosynthesis. J Biol Chem.

[33] Davidi L, Katz A, Pick U. Characterization of major lipid droplet proteins from Dunaliella. Planta. 2012:1–15. 10.1007/s00425-011-1585-7 22231009

[34] Tsai C-H, Warakanont J, Takeuchi T, Sears BB, Moellering ER, Benning C (2014). The protein compromised hydrolysis of Triacylglycerols 7 (CHT7) acts as a repressor of cellular quiescence in Chlamydomonas. Proc Natl Acad Sci U S A.

[35] Solovchenko AE, Merzlyak MN, Chivkunova OB, Reshetnikova IV, Khozina-Goldberg I, Didi-Cohen S, Cohen Z (2008). Effects of illumination and nitrogen starvation on accumulation of arachidonic acid by the microalga *Parietochloris incisa*. Mosc Univ Biol Sci Bull.

[36] Chen JE, Smith AG (2012). A look at diacylglycerol acyltransferases (DGATs) in algae. J Biotechnol.

[37] Miller R, Wu G, Deshpande RR, Vieler A, Gartner K, Li X, Moellering ER, Zauner S, Cornish AJ, Liu B (2010). Changes in transcript abundance in Chlamydomonas reinhardtii following nitrogen deprivation predict diversion of metabolism. Plant Physiol.

[38] Boyle NR, Page MD, Liu B, Blaby IK, Casero D, Kropat J, Cokus SJ, Hong-Hermesdorf A, Shaw J, Karpowicz SJ (2012). Three acyltransferases and nitrogen-responsive regulator are implicated in nitrogen starvation-induced triacylglycerol accumulation in Chlamydomonas. J Biol Chem.

[39] Gong Y, Zhang J, Guo X, Wan X, Liang Z, Hu CJ, Jiang M (2013). Identification and characterization of PtDGAT2B, an acyltransferase of the DGAT2 acyl-coenzyme a: diacylglycerol acyltransferase family in the diatom *Phaeodactylum tricornutum*. FEBS Lett.

[40] Zienkiewicz K, Zienkiewicz A, Poliner E, Du Z-Y, Vollheyde K, Herrfurth C, Marmon S, Farré EM, Feussner I, Benning C (2017). Nannochloropsis, a rich source of diacylglycerol acyltransferases for engineering of triacylglycerol content in different hosts. Biotechnol Biofuels.

[41] Liu J, Han D, Yoon K, Hu Q, Li Y (2016). Characterization of type 2 diacylglycerol acyltransferases in *Chlamydomonas reinhardtii* reveals their distinct substrate specificities and functions in triacylglycerol biosynthesis. Plant J.

[42] Stone Scot J., Levin Malin C., Farese Robert V. (2006). Membrane Topology and Identification of Key Functional Amino Acid Residues of Murine Acyl-CoA:Diacylglycerol Acyltransferase-2. Journal of Biological Chemistry.

[43] Wagner M, Hoppe K, Czabany T, Heilmann M, Daum G, Feussner I, Fulda M (2010). Identification and characterization of an acyl-CoA:diacylglycerol acyltransferase 2 (DGAT2) gene from the microalga *O. tauri*. Plant Physiol Biochem.

[44] Zhou X-R, Shrestha P, Yin F, Petrie JR, Singh SP (2013). AtDAGT2 is a functional acyl-CoA:diacylglycerol acyltransferase and displays different acyl-CoA substrate preferences than AtDGAT1. FEBS Lett.

[45] Wei H, Shi Y, Ma X, Pan Y, Hu H, Li Y, Luo M, Gerken H, Liu J (2017). A type-I diacylglycerol acyltransferase modulates triacylglycerol biosynthesis and fatty acid composition in the oleaginous microalga, Nannochloropsis oceanica. Biotechnol Biofuels.

[46] McFie PJ, Banman SL, Kary S, Stone SJ (2011). Murine diacylglycerol acyltransferase-2 (DGAT2) can catalyze triacylglycerol synthesis and promote lipid droplet formation independent of its localization to the endoplasmic reticulum. J Biol Chem.

[47] Yen Chi-Liang Eric, Stone Scot J., Koliwad Suneil, Harris Charles, Farese Robert V. (2008). Thematic Review Series: Glycerolipids.DGAT enzymes and triacylglycerol biosynthesis. Journal of Lipid Research.

[48] Xu N, Zhang SO, Cole RA, McKinney SA, Guo F, Haas JT, Bobba S, Farese RV, Mak HY (2012). The FATP1-DGAT2 complex facilitates lipid droplet expansion at the ER-lipid droplet interface. J Cell Biol.

[49] Herker E, Harris C, Hernandez C, Carpentier A, Kaehlcke K, Rosenberg AR, Farese RV, Ott M (2010). Efficient hepatitis C virus particle formation requires diacylglycerol acyltransferase 1 (DGAT1). Nat Med.

[50] Stanier RY, Kunisawa R, Mandel M, Cohen-Bazire G (1971). Purification and properties of unicellular blue-green algae (order *Chroococcales*). Bacteriol Rev.

[51] Sandager L, Gustavsson MH, Stahl U, Dahlqvist A, Wiberg E, Banas A, Lenman M, Ronne H, Stymne S (2002). Storage lipid synthesis is non-essential in yeast. J Biol Chem.

[52] Gietz RD, Schiestl RH (2007). High-efficiency yeast transformation using the LiAc/SS carrier DNA/PEG method. Nat Protoc.

[53] Lang I, Hodac L, Friedl T, Feussner I (2011). Fatty acid profiles and their distribution patterns in microalgae: a comprehensive analysis of more than 2000 strains from the SAG culture collection. BMC Plant Biol.

[54] Hagan IM (2016). Immunofluorescence microscopy of *Schizosaccharomyces pombe* using chemical fixation. Cold Spring Harb Protoc.

