# Supplementary material for: The type 2 acyl-CoA:diacylglycerol acyltransferase family of the oleaginous microalga Lobosphaera incisa
Source: BMC Plant Biol. 2018 Nov 26;18:298. doi: 10.1186/s12870-018-1510-3 (PMC6257963; doi:10.1186/s12870-018-1510-3)
Supplement: Supplementary file 2 — Alignment of amino acid sequences of L. incisa LiDGATs of type 2 with selected animal and plant DGAT2s and identification of conserved regions. (PDF 3602 kb) [file 12870_2018_1510_MOESM2_ESM.pdf]

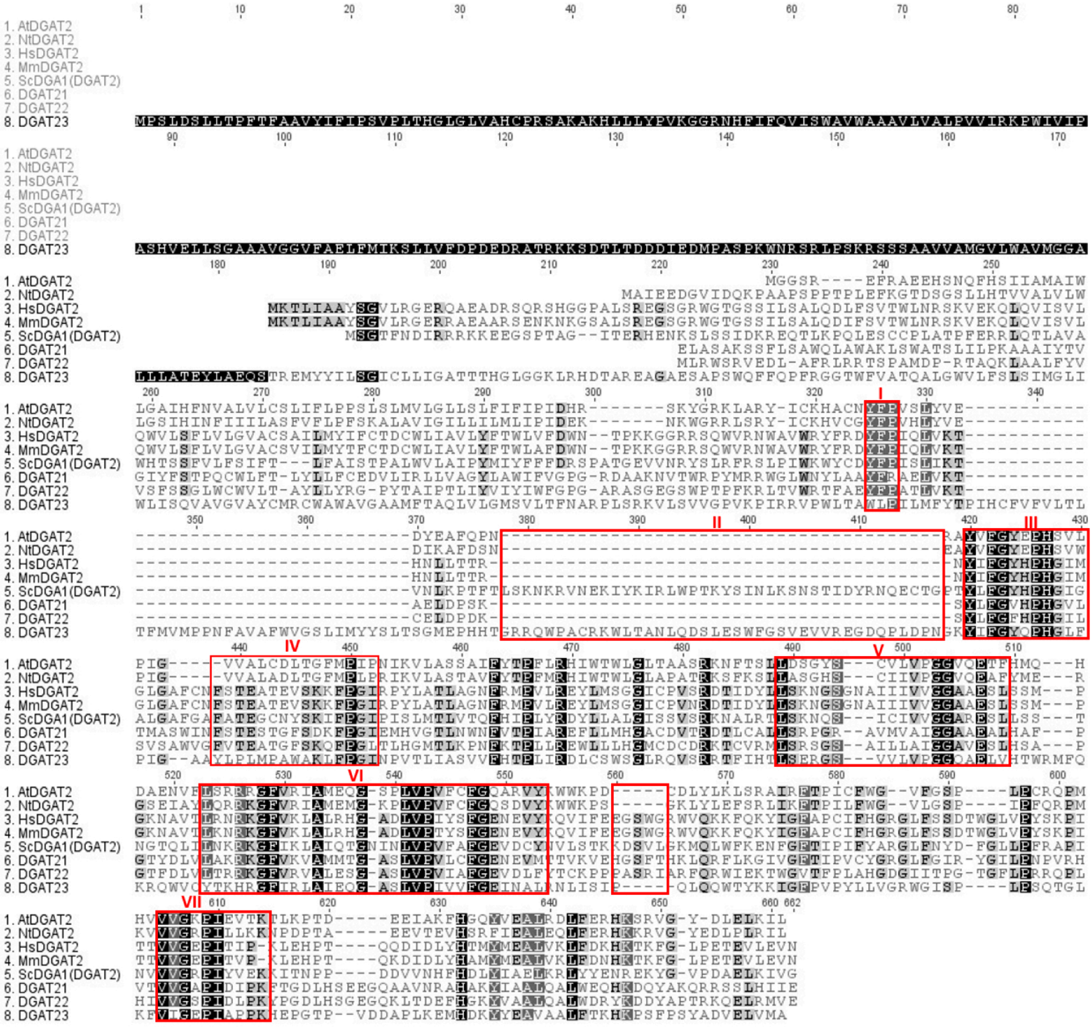
1. AtDGAT2
2. NtDGAT2
3. HsDGAT2
4. MmDGAT2
5. ScDGA1(DGAT2)
6. DGAT21
7. DGAT22
8. DGAT23
I
II
III
IV
V
VI
VII
