# Supplementary figures and images for: The type 2 acyl-CoA:diacylglycerol acyltransferase family of the oleaginous microalga Lobosphaera incisa
Source: BMC Plant Biol. 2018 Nov 26;18:298. doi: 10.1186/s12870-018-1510-3 (PMC6257963; doi:10.1186/s12870-018-1510-3)

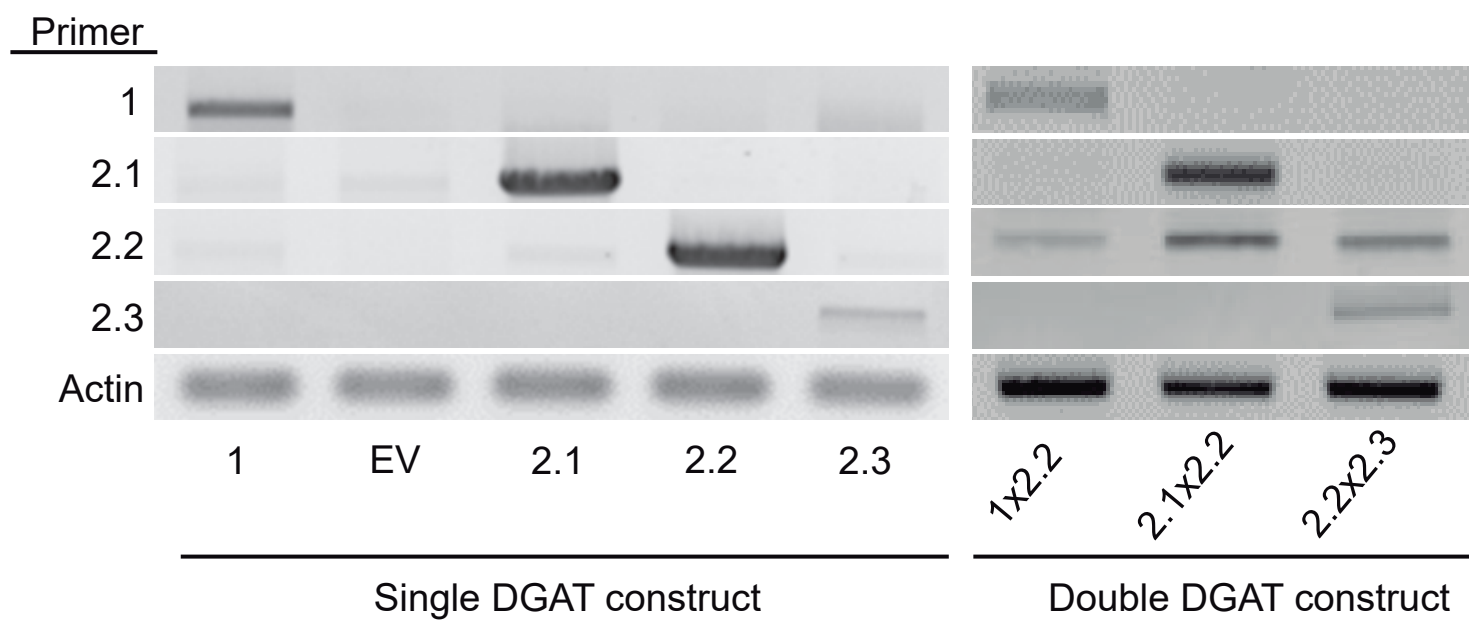

Supplement: Supplementary file 3 — Expression analysis of DGAT-encoding cDNAs in H1246 yeast mutant. (PDF 410 kb) [file 12870_2018_1510_MOESM3_ESM.pdf]

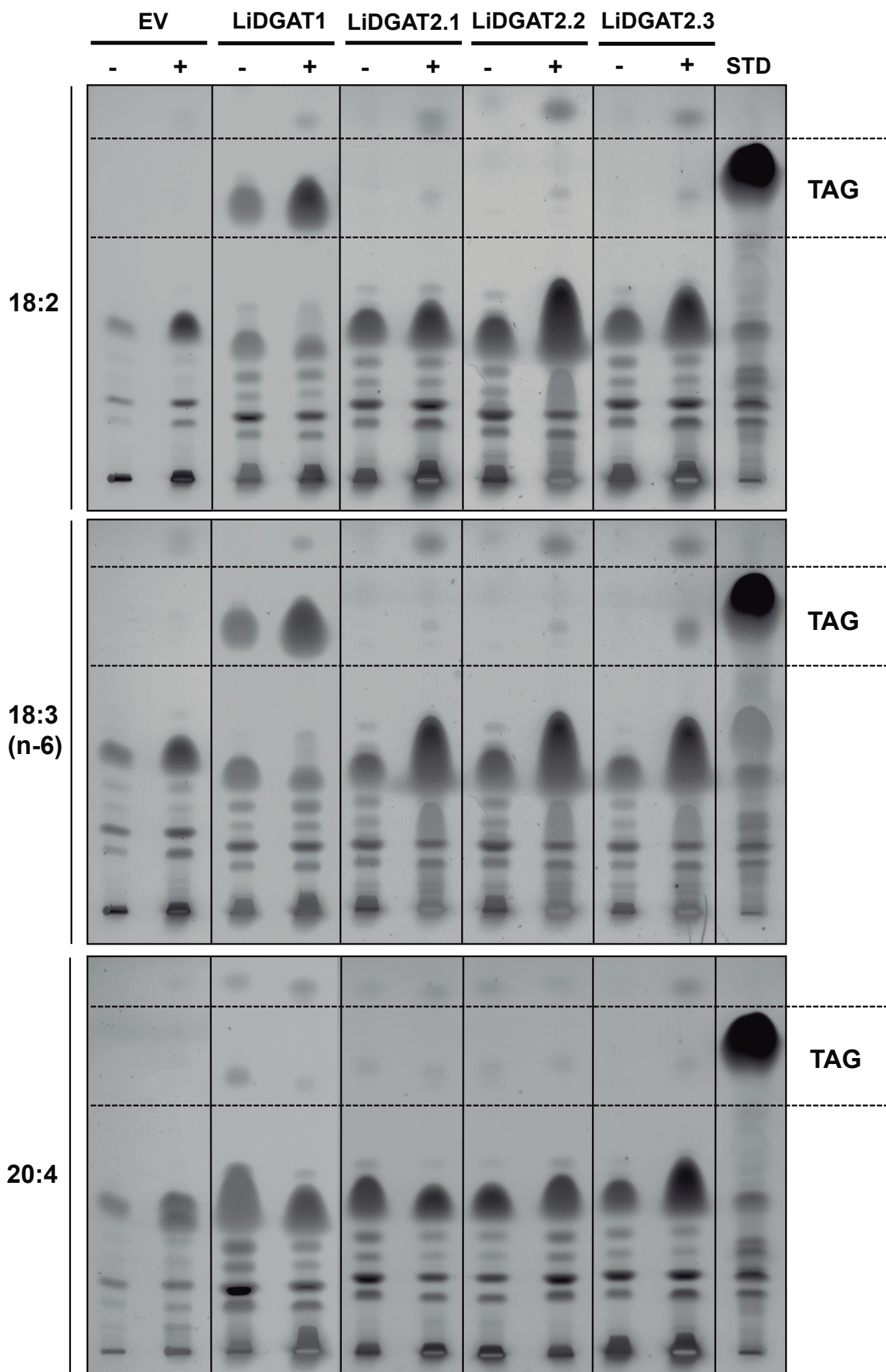

Supplement: Supplementary file 4 — Representative TLC plates showing activity of LiDGATs expressed in yeast mutants without and with feeding with exogenous FAs. TAG bands were used for lipid analysis showed in Fig. 4. (PDF 6144 kb) [file 12870_2018_1510_MOESM4_ESM.pdf]

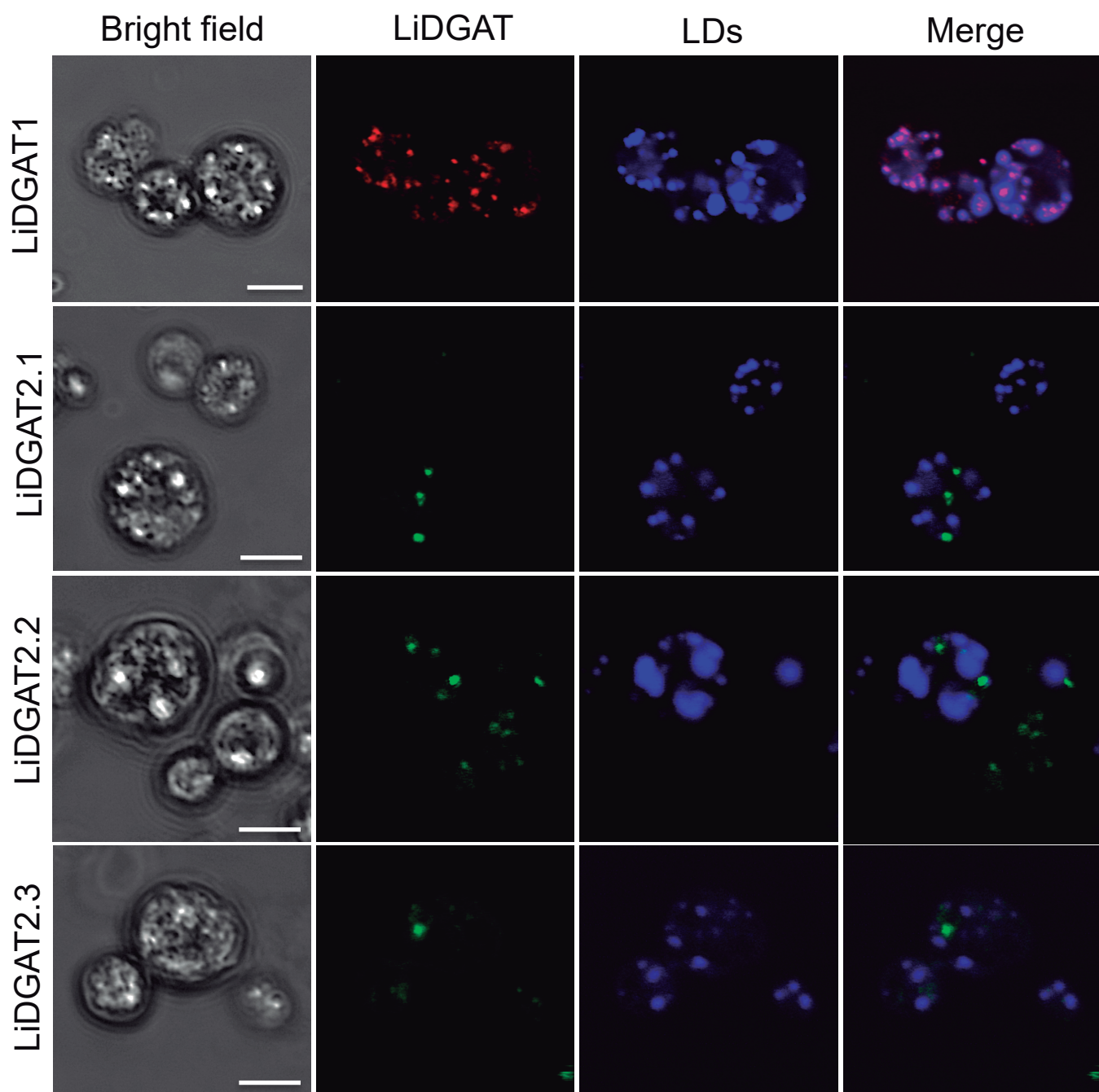

Supplement: Supplementary file 5 — Immunodetection of LiDGAT1 (red), three type 2 LiDGATs (green) and LDs (blue) in H1246 cells complemented with the single constructs. Anti-myc antibody and anti-FLAG antibody were used for detection of LiDGAT1 and each of LiDGAT2, respectively. Bar = 5 μm. (PDF 12506 kb) [file 12870_2018_1510_MOESM5_ESM.pdf]

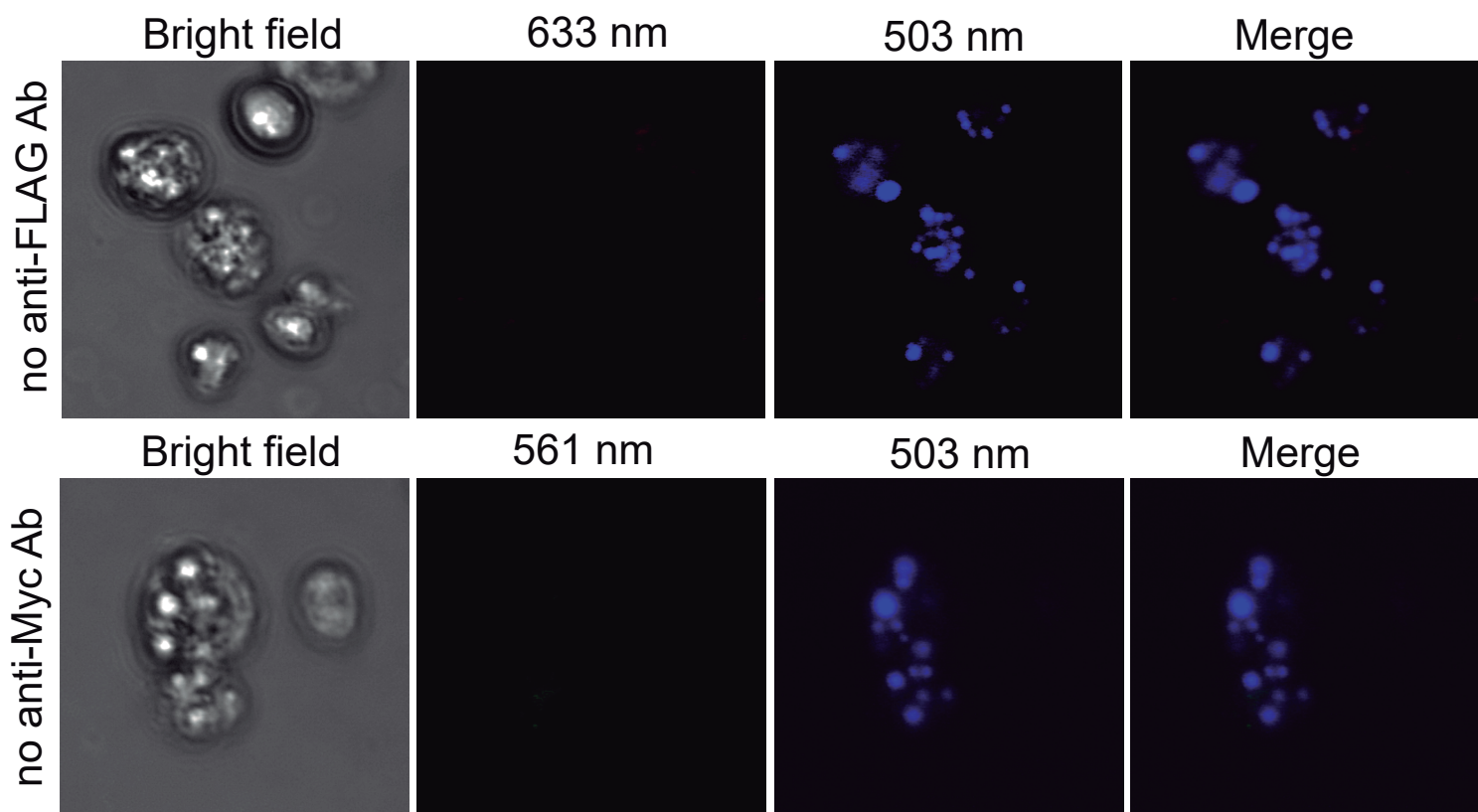

Supplement: Supplementary file 6 — Control reaction of immunodetection of LiDGATs performed with omission of the primary antibodies. No fluorescence corresponding to LiDGAT1 at 633 nm or LiDGAT2.2 at 561 nm can be observed. Labelled LDs are shown in blue. (PDF 4265 kb) [file 12870_2018_1510_MOESM6_ESM.pdf]
