# Supplementary material for: The type 2 acyl-CoA:diacylglycerol acyltransferase family of the oleaginous microalga Lobosphaera incisa
Source: BMC Plant Biol. 2018 Nov 26;18:298. doi: 10.1186/s12870-018-1510-3 (PMC6257963; doi:10.1186/s12870-018-1510-3)
Supplement: Supplementary file 7 — Table listing DGAT-encoding genes used for phylogenetic and sequence analyses. (PDF 151 kb) [file 12870_2018_1510_MOESM7_ESM.pdf]

Gene	GeneBank accession no.	Organism
LiDGAT1	MF576159	*Lobosphaera incisa*
LiDGAT2.1	MH290880	*Lobosphaera incisa*
LiDGAT2.2	MH290881	*Lobosphaera incisa*
LiDGAT2.3	MH290882	*Lobosphaera incisa*
AtDGAT1	AEC06882.1	*Arabidopsis thaliana*
ApDGAT1	KFM28983.1	*Auxenchlorella protothecoides*
BnDGAT1	AFM31262.1	*Brassica napus*
HsDGAT1	AAI50650.1	*Homo sapiens*
Pt1090DGAT1	ADY76581.1	*Phaeodactylum tricornutum*
MmDGAT1	EDL29575.1	*Mus musculus*
TpDGAT1	ADV58933.2	*Thalassiosira pseudonana*
AtDGAT2	AEE78802.1	*Arabidopsis thaliana*
CrDGTT1	AFB73929.1	*Chlamydomonas rheinhardtii*
HsDGAT2	AIC52594.1	*Homo sapiens*
MmDGAT2	EDL16367.1	*Mus musculus*
NtDGAT2	AGL46984.1	*Nicotiana tabacum*
Pt9DGAT2A	AFQ23660.1	*Phaeodactylum tricornutum*
Pt9DGAT2B	AFM37314.1	*Phaeodactylum tricornutum*
Pt9DGAT2C	AFQ23659.1	*Phaeodactylum tricornutum*
ScDGA1	KQC40924.1	*Saccharomyces cerevisiae*
AhDGAT3	AAX62735.1	*Arachis hypogaea*
VfDGAT3	AGL81309.1	*Vernicia fordii*
Ac(ADP1)1WS/DGAT	AGE15444.1	*Actinobacter calcoaceticus*
