# Supplementary material for: The type 2 acyl-CoA:diacylglycerol acyltransferase family of the oleaginous microalga Lobosphaera incisa
Source: BMC Plant Biol. 2018 Nov 26;18:298. doi: 10.1186/s12870-018-1510-3 (PMC6257963; doi:10.1186/s12870-018-1510-3)
Supplement: Supplementary file 9 — List of the vectors used in this work for yeast transformation. (PDF 95 kb) [file 12870_2018_1510_MOESM9_ESM.pdf]

A

Primer	5'-3' Sequence
LiDGAT1$_{opt}$F-BamHI	ATAGGATCCATGTCGGGCATGTCTGGC
LiDGAT1$_{optNS}$R-XhoI	ATACTCGAGTCACCGGTTGAGGACGCA
LiDGAt2.1$_{opt}$F-BamHI	ATAGGATCCATGGAATTGGCCTCAGCTAAG
LiDGAt2.1$_{optNS}$R-SalI	ATAGTCGACCTCGATGATGTGCAGGCTG
LiDGAt2.2$_{opt}$F-BamHI	ATAGGATCCATGGCTTTGAGATGGTCTAGAGT
LiDGAt2.2$_{optNS}$R-SalI	ATAGTCGACCTCGACCATGCGCAGCTC
LiDGAt2.3$_{opt}$F-BamHI	ATAGGATCCATGGCTCCATCTTTGGACTCT
LiDGAt2.3$_{optNS}$R-SalI	ATAGTCGACAGCCATGACGAGCTCCAC

OPT; codon optimized, NS; no stop codon.

Underlined - restriction sites.

B

Backbone	MCS1	MCS2	Reference
pEsc-Ura	Empty	Empty	Agilent technologies Cat no. 217454-51
pEsc-Ura	LiDGAT1opt-FLAG	Empty	*This work*
pEsc-Ura	Empty	LiDGAT2.1opt-myc	*This work*
pEsc-Ura	Empty	LiDGAT2.2opt-myc	*This work*
pEsc-Ura	Empty	LiDGAT2.3opt-myc	*This work*
pEsc-Ura	LiDGAT1opt-FLAG	LiDGAT2.2opt-myc	*This work*
pEsc-Ura	LiDGAT2.1opt-FLAG	LiDGAT2.2opt-myc	*This work*
pEsc-Ura	LiDGAT2.2opt-FLAG	LiDGAT2.3opt-myc	*This work*

MCS; multiple clonning site.
